# NF-κB p65 dimerization and DNA-binding is important for inflammatory gene expression

**DOI:** 10.1096/fj.201801638R

**Published:** 2018-12-07

**Authors:** Tabea Riedlinger, Robert Liefke, Johanna Meier-Soelch, Liane Jurida, Andrea Nist, Thorsten Stiewe, Michael Kracht, M. Lienhard Schmitz

**Affiliations:** * Institute of Biochemistry, Member of the German Center for Lung Research, Justus-Liebig-University, Giessen, Germany;; † Institute of Molecular Biology and Tumor Research (IMT), Philipps University Marburg, Marburg, Germany;; ‡ Rudolf-Buchheim–Institute of Pharmacology, Member of the German Center for Lung Research, Justus-Liebig-University, Giessen, Germany; and; § Genomics Core Facility–Institute of Molecular Oncology, Philipps University Marburg, Marburg, Germany

**Keywords:** inflammation, chromatin, transcription

## Abstract

Increasing evidence shows that many transcription factors execute important biologic functions independent from their DNA-binding capacity. The NF-κB p65 (RELA) subunit is a central regulator of innate immunity. Here, we investigated the relative functional contribution of p65 DNA-binding and dimerization in p65-deficient human and murine cells reconstituted with single amino acid mutants preventing either DNA-binding (p65 E/I) or dimerization (p65 FL/DD). DNA-binding of p65 was required for RelB-dependent stabilization of the NF-κB p100 protein. The antiapoptotic function of p65 and expression of the majority of TNF-α–induced genes were dependent on p65’s ability to bind DNA and to dimerize. Chromatin immunoprecipitation with massively parallel DNA sequencing experiments revealed that impaired DNA-binding and dimerization strongly diminish the chromatin association of p65. However, there were also p65-independent TNF-α–inducible genes and a subgroup of p65 binding sites still allowed some residual chromatin association of the mutants. These sites were enriched in activator protein 1 (AP-1) binding motifs and showed increased chromatin accessibility and basal transcription. This suggests a mechanism of assisted p65 chromatin association that can be in part facilitated by chromatin priming and cooperativity with other transcription factors such as AP-1.—Riedlinger, T., Liefke, R., Meier-Soelch, J., Jurida, L., Nist, A., Stiewe, T., Kracht, M., Schmitz, M. L. NF-κB p65 dimerization and DNA-binding is important for inflammatory gene expression.

The family of NF-κB transcription factors (TFs) consists of 5 different DNA-binding subunits, namely p65 (RELA), c-Rel (REL), RelB, p105/p50 (NF-κB1), and p100/p52 (NF-κB2) (1). The p65, c-Rel and RelB subunits contain C-terminal transactivation domains, which allow the induction of gene expression (1). NF-κB is a dimeric and inducible TF that is activated in response to a plethora of different stimuli representing adverse physiologic conditions including infection and inflammation. The key step in its regulation is the generation of free DNA-binding subunits, which are trapped in the cytosol of unstimulated cells by association with inhibitory IκB proteins including IκBα, IκBβ, IκBε, and the p105/p100 precursor proteins (2, 3). The liberation of DNA-binding subunits occurs by 2 alternative mechanisms. In the canonical activation pathway, IκB proteins are inducibly phosphorylated, followed by their ubiquitin-proteasome–mediated degradation and the release of free DNA-binding subunits (4). Alternatively, the DNA-binding subunits are generated *via* processing of p100 and p105 to yield the active DNA-binding forms p52 and p50, respectively (5). Precursor processing occurs either during translation or through phosphorylation-induced partial proteasome-dependent proteolysis to control the physiologic homeostasis of NF-κB signaling (6–9).

The activity of the DNA-binding subunits is further regulated by multiple posttranslational modifications (PTMs) that affect protein stability and protein-protein interactions and also the DNA-binding capacity of NF-κB. An example for the latter mechanisms is the acetylation of p65 at Lys 221, which causes a conformation change that favors NF-κB DNA-binding (10). The DNA-binding activity can be inhibited upon nitration of Tyr 66 and Tyr 152, asymmetric dimethylation of Arg 30, or phosphorylation at Ser 42/45 (11–13). Regulation of NF-κB DNA-binding is not only mediated by PTMs but also by some NF-κB inhibiting agents such as sesquiterpene lactones (14).

DNA-bound NF-κB can interact with many TFs to orchestrate the timing and amplitude of gene expression. In addition, NF-κB binds to histone acetyl transferases such as cAMP-response element binding protein (CREB) binding protein (CBP)/p300 in a promoter-specific fashion, which in turn allows deposition of the enhancer mark H3K27Ac to trigger expression of genes regulating the response to infections, inflammation, and cell survival (15). One of the NF-κB target genes is IκBα, which is resynthesized after its inducible degradation and serves to remove NF-κB from its cognate DNA to shut down NF-κB activity as part of an autoregulatory feedback loop (16). Although it is well known that the DNA-binding capacity of NF-κB is regulated, the physiologic consequences of absent NF-κB DNA-binding and/or homodimerization have not been studied. A large number of TFs including ERα, E2F-1, Hand2, TAL-1, and SCL show DNA-binding independent functions (17–21). Inactivation of the DNA-binding function of the tumor suppressor p53 can even lead to gain-of-function phenotypes (22). NF-κB DNA-binding can be regulated under physiologic conditions by PTMs and in pathophysiological situations by drugs or mutations (23), but the contribution of DNA-binding for the cellular functions of NF-κB is not yet known. It was therefore interesting to investigate the role of NF-κB DNA-binding and dimerization for its function *in vivo*.

To address this question in a physiologic setting, we generated human HeLa cells and mouse embryonic fibroblasts (MEFs) expressing p65 proteins with defects in these functions. Both DNA-binding and dimerization are important for the antiapoptotic function of NF-κB and TNF-α–induced gene expression. Chromatin immunoprecipitation (ChIP) with massively parallel DNA sequencing (ChIP-seq) experiments demonstrated a globally reduced chromatin association of the mutants but also allowed to discriminate between direct and assisted binding, as defined by genomic loci allowing residual inducible chromatin association of DNA-binding defective p65. These loci were enriched in activator protein 1 (AP-1) binding sites and also showed increased baseline chromatin accessibility and transcription, pointing to the relevance of functional interactions and cooperativity between different TFs and an accessible chromatin status. The functional interplay between AP-1 and NF-κB sites was revealed after mutation of binding sites for these TFs in the *IL-8* promoter. These experiments also unraveled 2 new regulatory circuits controlling subunit abundance within the NF-κB system because p65 DNA-binding and dimerization is important for expression of the NF-κB subunit RelB, which in turn leads to the stabilization of the p52 precursor protein p100. In addition, we observed a rapid decay of the free dimerization-deficient NF-κB p65 subunit to ensure the balanced subunit stoichiometry of NF-κB complexes.

## MATERIALS AND METHODS

### Cell culture and generation of stable cell lines

HeLa and MEF cells were cultured in DMEM (Thermo Fisher Scientific, Waltham, MA, USA) supplemented with fetal calf serum and penicillin-streptomycin. For clustered regularly interspaced short palindromic repeat (CRISPR-Cas9)–mediated knockout of p65, 1000 HeLa cells per 10-cm dish were transfected with 6 µg of the pX459 vector containing a single guide RNA targeting the third exon of p65. After 1 d, the nontransfected cells were eliminated by the addition of puromycin (1 µg/ml) for 48 h. After ∼1 wk, single cell-derived clones were picked and further analyzed for expression of p65 and Cas9. Cell lines established from single cell-derived p65-deficient clones were reconstituted by transfection with plasmids encoding either wild-type (WT) or mutant p65 or the empty vector control. Following selection of stable cell clones with puromycin for 2 wk, the individual clones were picked and analyzed for appropriate p65 expression and NF-κB signaling. The reconstituted HeLa p65 knockout clone #10 cells were used for all experiments, but all main findings were recapitulated in different cell clones to ensure that the findings are not confined to individual cell lines.

### Antibodies, plasmids, oligonucleotides, and reagents

This information is provided in Supplemental Table S1.

### Cell fractionation, protein extraction, and Western blotting

Whole cell lysates were prepared either by lysing the cells directly in 1 time SDS sample buffer or by lysing the cells in a buffer containing the nonionic detergent NP-40 [20 mM Tris-HCl pH 7.5, 150 mM NaCl, 1 mM PMSF, 10 mM NaF, 0.5 mM sodium orthovanadate, leupeptine (10 µg/ml), aprotinin (10 µg/ml), 1% NP-40 and 10% glycerol] as previously described (24). Cell fractionation into cytoplasmic, nuclear, and the chromatin fraction was done as previously published (25). Proteins were loaded on denaturing Tris-HCl polyacrylamide gels, and the samples were transferred to a PVDF membrane by semidry blotting. All bands were visualized using the ChemiDocTM XRS+ System (Bio-Rad Laboratories, Lunteren, The Netherlands). Image Lab software (Bio-Rad Laboratories) was used for quantification of chemiluminescence intensity.

### Cell viability

Cell viability assays were conducted by seeding cells of the various MEF cell lines on d 0. One day later, the medium was replaced by fresh DMEM medium containing 0, 5, 10, 15, or 20 ng/ml mouse TNF-α. After 24 h, cells were harvested with trypsin and cell viability was measured using a Casy Cell Counter and Analyzer (Schärfe Systems, Reutlingen, Germany) Model TTC. Relative cell numbers were calculated by setting the number of viable cells not receiving any mouse TNF-α to 100% for each MEF line.

### Real-time quantitative PCR

Total RNA was isolated from cells using the RNeasy Kit according to the procedure described by the manufacturer (Qiagen, Hilden, Germany). One microgram of RNA was used for the generation of cDNA using Oligo(dT)12–18 Primers and SuperScript II Reverse Transcriptase (Thermo Fisher Scientific). cDNA was diluted and used for the quantitative PCR reactions using SYBR green as a reporter. Every reaction was performed as duplicates and quantified with the ΔΔ*C_t_* method. Therefore, threshold cycles (*C_t_*) of target genes were normalized to a housekeeping gene (*TPI*). The resulting Δ*C_t_* were compared to control samples, and relative mRNA expression was calculated by *R* = 2^−ΔΔ*Ct*^.

### EMSA

Lysates were prepared with Totex buffer [20 mM Hepes/KOH (pH 7.9); 350 mM NaCl; 20% (v/v) glycerol; 1% (v/v) NP-40; 1 mM MgCl_2_; 0.5 mM EDTA; 0.1 mM EGTA; freshly added 0.5 mM Na_3_VO_4_; 10 mM NaF; 1 mM PMSF]. Equal amounts of protein contained in lysates were mixed with poly (dI-dC), bovine serum albumin, [^32^P]-labeled oligonucleotide encompassing a κB site, and 5 times EMSA-binding buffer [100 mM Hepes/KOH (pH 7.9; 300 mM NaCl; 1 mM DTT; 20% [v/v] Ficoll 400]. The mixture was incubated at room temperature for 30 min and subsequently loaded onto a 4% native polyacrylamide gel. After the run, the gel was dried and exposed to an X-ray film for 24 h at −80°C.

### ChIP and ChIP-seq

Twenty-four hours prior to the experiment, 1 × 10^7^ HeLa were seeded onto T175 flasks. The next day, cells were treated for 0 or 60 min with TNF-α (20 ng/ml) and cross-linked with formaldehyde (1% final concentration) for 10 min, followed by addition of glycine (100 mM final concentration) for 5 min. Cells were collected in the medium using a cell scraper and immediately put on ice. Two flasks from the same condition were pooled. After centrifugation for 5 min at 4°C and 3000 rpm, supernatant was aspirated, and pellet was resuspended in 2 ml ice cold PBS with PMSF (0.5 mM final concentration). Following centrifugation for 5 min at 4°C and 3000 rpm, supernatant was aspirated, and cells were resuspended in ice cold lysis-buffer [1% SDS, 10 mM EDTA, 50 mM Tris-HCl pH 8.1, 0.5 mM PMSF, Complete Protease Inhibitor Cocktail (MilliporeSigma, Burlington, MA, USA)]. Lysis took place on ice for 10 min. One milliliter of the lysate was transferred to Bioruptor tubes and sonicated in a Bioruptor (Diagenode, Denville, NJ, USA) with the following program: 30 s on, 30 s off, power high, 28 cycles. Sonicated lysates were centrifuged for 15 min at 13,200 rpm at 4°C and supernatants transferred to new reaction tubes. Aliquots representing 25 µg of chromatin were diluted 1:10 with dilution buffer (0.01% SDS, 1.1% Triton X-100, 1.2 mM EDTA, 167 mM NaCl, 16,7 mM Tris-HCl pH 8.1) and subjected to 4 h of preclearing with an agarose A/G-bead mixture and 2 µg rabbit IgG antibody at 4°C with end-over-end tumbling. Supernatants were incubated with either a rabbit pAb against the C-terminal domain of the NF-κB subunit p65 (C-20) or rabbit IgG antibody. Immunoprecipitation was carried out overnight with end-over-end tumbling at 4°C. A mixture of agarose A/G-beads was added for 4 h, afterwards immunoprecipitated complexes were successively washed for 5 min at 4°C with end-over-end tumbling with low-salt buffer (0.1% SDS, 1% Triton X-100, 2 mM EDTA, 150 mM NaCl, 20 mM Tris-HCl pH 8.1), high-salt buffer (0.1% SDS, 1% Triton X-100, 2 mM EDTA, 500 mM NaCl, 20 mM Tris-HCl pH 8.1), LiCl-buffer (250 mM LiCl, 1% NP40, 1% Deoxycholat, 1 mM EDTA, 10 mM Tris-HCl pH 8.1), and twice with TE-buffer (10 mM Tris-HCl pH 8.1, 1 mM EDTA). Reverse-crosslinking took place in TE-buffer with addition of RNase A for 30 min and Proteinase K for 2 h at 37°C followed by incubation at 65°C overnight. Free DNA was purified using NucleoSpin Gel and PCR Clean Up Kit with NucleoTrap buffer (Macherey-Nagel, Düren, Germany) and was eluted in 50 µl Elution buffer or nuclease-free H_2_O (for ChIP-seq). ChIP-seq libraries were prepared from purified chromatin immunoprecipitation DNA with the Microplex Library Preparation Kit (Diagenode) according to the manufacturer’s instructions. The quality of sequencing libraries was controlled on a Bioanalyzer 2100 using the Agilent High Sensitivity DNA Kit (Agilent Technologies, Santa Clara, CA, USA). Pooled sequencing libraries were quantified with digital PCR (QuantStudio 3D; Thermo Fisher Scientific) and sequenced on the HiSeq 1500 platform (Illumina, San Diego, CA, USA) in Rapid-Run mode with 50 bases single reads.

### Luciferase reporter gene assays

Cells were seeded in 6-well plates, and on the next day they were transfected with 1 µg of a firefly reporter plasmid together with a plasmid-encoding *Renilla* luciferase. Transfected cells were stimulated 2 d later with TNF-α, and luciferase activity was determined by using the Dual Luciferase Assay Kit from Promega (Madison, WI, USA). The emitted bioluminescence was detected with a Berthold Lumat LB 9507 luminometer. The relative activities were calculated after the normalization of the firefly luciferase activities to the activities of the *Renilla* luciferase.

### RNA-sequencing

The differentially reconstituted HeLa cells and MEFs were left untreated or stimulated with TNF-α (20 ng/ml) for 60 min as specified in the figure legends. Cells were harvested and total RNA was isolated by using the NucleoSpin RNA Kit (Macherey-Nagel). Prior to library preparation, RNA quality was assessed by using the Experion RNA StdSens Analysis Kit (Bio-Rad Laboratories BV). RNA-sequencing (RNA-seq) libraries were prepared from total RNA by using the TruSeq Stranded total RNA LT Kit (Illumina) according to the manufacturer’s instructions. Quality of sequencing libraries was controlled on a Bioanalyzer 2100 by using the Agilent High Sensitivity DNA Kit (Agilent Technologies). Pooled sequencing libraries were quantified with digital PCR (QuantStudio 3D; Thermo Fisher Scientific) and sequenced on the HiSeq 1500 platform (Illumina) in Rapid-Run mode with 50 bases single reads.

### Bioinformatic analysis

RNA-seq data were processed in Galaxy (26). The data were mapped to human genome hg38 by using RNA STAR (27), and normalized gene expression data were obtained *via* Cufflinks (28) by using a gffread-filtered gencode.v27.basic.annotation.gtf file as the template. Principal component analysis (PCA) was performed by using the Bioconductor package prcomp using the top 100 variable genes. Analysis of the ChIP-seq data was performed by using Galaxy/Cistrome and Bioconductor (29, 30). ChIP-seq data were aligned to human genome hg19 by using bowtie 1.0 (31) with *n* = 1 and *m* = 3 as parameter. Normalized bigwig files were obtained by using a Model-based analysis of ChIP-Seq (MACS) data within the Galaxy/Cistrome platform. Significant peaks were identified by using MACS 1.0. To identify the genes related to called peaks, the GREAT tool (32) was used. Motif analysis was performed by using i-cisTarget using curated motif databases (33). Promoters were defined as region from −1000 to +1000 base pairs (bp) relative to the transcription start site. Kyoto Encyclopedia of Genes and Genomes (KEGG) analysis was performed using the Database for Annotation, Visualization and Integrated Discovery (DAVID) (34). The following publicly available data sets were used: c-Jun (GSM935341), JunD (GSM935328), c-Fos (GSM935317), DNase I Hypersensitivity (GSM816643) (35), H3K27ac−TNF-α (GSM1305204), H3K27ac +TNF-α (GSM1305205), BRD4−TNF-α (GSM1305201), BRD4 +TNF-α (GSM1305202), RNA Polymerase II−TNF-α (GSM1305213), RNA Polymerase II +TNF-α (GSM1305214) (36).

### Data and materials

The data generated in this study are available at the Gene Expression Omnibus repository (National Center for Biotechnology Information, Bethesda, MD, USA; *https://www.ncbi.nlm.nih.gov/geo/*), Accession No. GSE116284.

## RESULTS

### Functional characterization of cells expressing p65 DNA-binding and dimerization-defective variants

**Figure 1:**
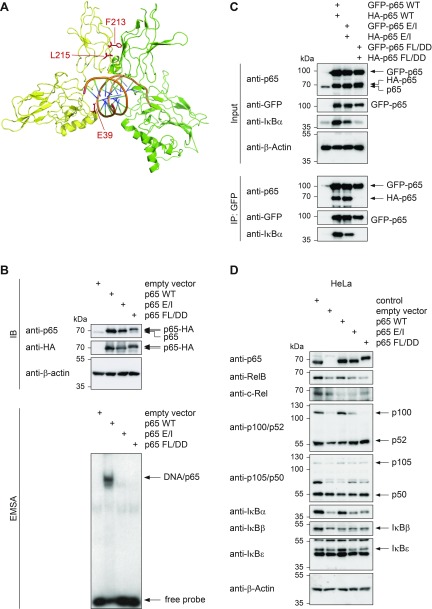
Generation and characterization of p65 mutant cell lines. *A*) The dimer between p65 (yellow) and p50 (green) in complex with DNA is shown (Protein Data Bank: 1vkx) (64). The Pymol program was used to highlight the mutated amino acids in red. *B*) HA-tagged WT p65, p65 E/I, or p65 FL/DD were expressed in HEK-293T cells. Whole cell extracts were prepared and tested by immunoblot (IB) with anti-HA antibodies to ensure the correct and comparable expression of HA-tagged proteins (upper). Another aliquot was used for an EMSA experiment, the autoradiogram shows the positions of free and p65-bound [^32^P]-labeled oligonucleotides (lower). *C*) HEK293T cells were transfected to express green fluorescent protein–tagged and HA-tagged p65 in the WT or mutated forms as shown. Cell extracts were generated and used for Western blotting (upper) or for coimmunoprecipitation experiments (lower) as shown. The positions of the detected proteins are indicated by arrows, MW markers are given. *D*) HeLa cells lacking p65 expression after CRISPR-Cas9–mediated gene editing were used for the generation of stable cell lines expressing physiologic amounts of p65 and its mutants fused to a C-terminal HA-tag. Expression of p65 and other NF-κB regulatory proteins was assessed by immunoblotting with the indicated antibodies.

In order to investigate the function of p65 DNA-binding and dimerization in different species and cell types, we performed reconstitution experiments in MEFs derived from *RELA*-deficient mice (37) and in human HeLa cells where p65 protein expression was eliminated by CRISPR-Cas9 (Supplemental Fig. S1*A*). Various p65-deficient HeLa cell clones were tested for NF-κB signaling. Whereas the upstream signaling steps leading to IκBα-degradation were still functional (Supplemental Fig. S1*B*), p65-deficient clones showed absent expression of well-characterized NF-κB target genes (Supplemental Fig. S1*C*). Both MEF and HeLa p65-deficient cells were used to reconstitute expression of p65, either with the WT form or with mutants defective in DNA-binding or dimerization. To create a DNA-binding defective mutant, we exchanged Glu 39, a residue known to be important for DNA-binding (38), to Ile. A dimerization-defective mutant was generated by simultaneous mutation of Phe 213 and Leu 215 to Asp (39). The relative positions of the mutated amino acids within the p65 protein are shown in **Fig. 1*A*** and Supplemental Fig. S1*D*. EMSAs showed that the p65 E/I mutant and also the p65 FL/DD mutant had lost their ability to bind to an oligonucleotide containing a NF-κB site (Fig. 1*B*), consistent with the reported necessity of p65 dimerization for DNA-binding (40). Defective dimerization of the p65 FL/DD mutants was confirmed by coimmunoprecipitation experiments in cells expressing green fluorescent protein–tagged and hemagglutinin (HA)-tagged p65 FL/DD proteins (Fig. 1*C* and Supplemental Fig. S1*E*). To test the role of p65 dimerization and DNA-binding *in vivo*, we used the p65-deficient HeLa and MEF cells to stably reconstitute the expression of p65 WT, p65 E/I, and p65 FL/DD. As a control, HeLa WT cells (control) were always subjected to the same conditions as the differentially reconstituted CRISPR-Cas9–mediated p65 knockout HeLa cells. HeLa and MEF cell clones expressing p65 amounts comparable to those contained in WT control cells showed unchanged proliferation (Supplemental Fig. S2*A*) and were further analyzed for the expression of other NF-κB DNA-binding subunits and IκB proteins. Western blotting ensured comparable expression of p65, although the p65 FL/DD mutant consistently migrated slightly slower than the other p65 variants in SDS gels (Fig. 1*D*). This unusual electrophoretic behavior of p65 occurred in several reconstituted HeLa cell clones (Fig. 1*D*) and MEFs (Supplemental Fig. S2*B*, *C*), a feature that is often seen for p65 point mutants due to the intrinsic conformational flexibility of this molecule (41). Also, the expression of IκBα was reduced in p65-deficient cells as well as in p65 E/I and p65 FL/DD expressing cells, consistent with its role as an NF-κB target gene (42). Interestingly, these cells also showed impaired expression of RelB and a very strong reduction of p100 protein levels in HeLa cells (Fig. 1*D*) and MEFs (Supplemental Fig. S2*C*), although the p100 mRNA levels remained unchanged (data not shown).

**Figure 2:**
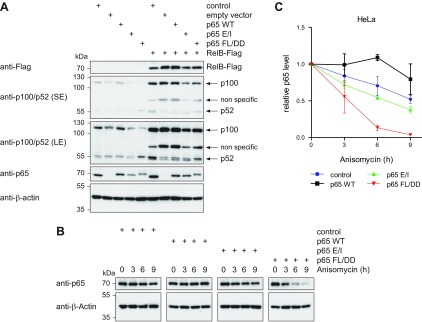
Functional analysis of p65 FL/DD stability and diminished p100 expression in p65-deficient cells. *A*) The indicated HeLa cell lines were transfected to express Flag-tagged RelB as shown and analyzed for protein expression by immunoblotting. The expression of p100/p52 is shown in a short exposure (SE) and long exposure (LE). The unspecific recognition of RelB by the p100/p52 antibody is indicated. *B*) The stability of p65 and its mutant versions under steady state conditions was tested by blocking protein synthesis with anisomycin (2.5 µg/ml) for various time periods, followed by analysis of p65 levels *via* immunoblotting. *C*) Quantification of *B*, all values were normalized to the β-actin levels, untreated cells were arbitrarily set as 1. Error bars represent sem from 3 independent experiments.

These data suggest that processing of p100 to p52 depends on the presence and function of the p65 protein. To test whether the decreased p100 levels are attributable to an increased proteasomal processing of p100, p65-deficient cells were incubated in the presence of the proteasome inhibitor lactacystin and analyzed for p100/p52 levels. These experiments did not reveal any contribution of the ubiquitin-proteasome system for the reduced p100 levels (Supplemental Fig. S3*A*), suggesting the occurrence of another mechanism. Because p100 processing can also be regulated by RelB binding to p100 (43), we transiently rescued RelB expression in the different HeLa cell lines and observed a robust increase in p100/p52 expression to similar levels (**Fig. 2*A***), suggesting that the p65-dependent regulation of p100 stability is indirectly mediated by RelB.

During the screening and characterization of the cell clones expressing reconstituted NF-κB p65 we made the serendipitous observation that many clones expressing p65 FL/DD showed frequently lower p65 expression levels. To investigate whether this is due to reduced protein stability, protein synthesis was blocked with anisomycin and constitutive p65 decay was monitored over time in reconstituted HeLa cells (Fig. 2*B, C*) and MEFs (Supplemental Fig. S3*B*). These experiments showed a strongly decreased stability of the dimerization-defective p65 FL/DD protein in both cell systems, suggesting that free and monomeric p65 undergoes rapid degradation. This mechanism might be used to ensure the balanced subunit stoichiometry of NF-κB complexes and similar mechanisms occur in a number of multiprotein complexes (44, 45).

### p65 DNA-binding and dimerization affect the dynamics of subcellular distribution and regulation of TNF-α–induced negative feedback loops

**Figure 3:**
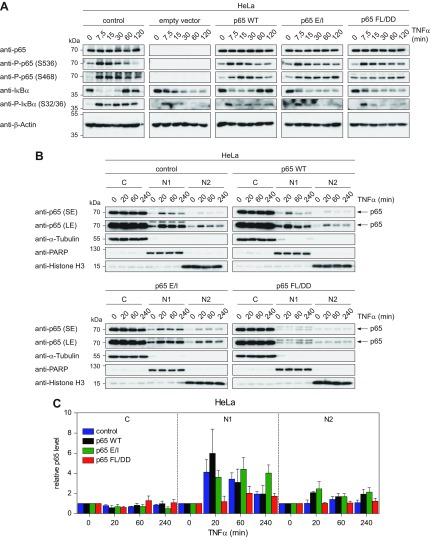
Analysis of differentially reconstituted HeLa cells for changes in dynamic NF-κB signaling. *A*) The reconstituted HeLa cells were stimulated for different periods with TNF-α, and stability and phosphorylation of p65 and IκBα was analyzed by immunoblotting, a representative experiment is displayed. *B*) The differentially reconstituted HeLa cells were treated for the indicated periods with TNF-α and cells were fractionated into the cytosolic (C), soluble nuclear (N1) and insoluble nuclear (N2) fractions. These fractions were analyzed for the kinetics of p65 nuclear import and export by immunoblotting. The purity of the fractions was controlled by blotting for tubulin (C), PARP (N1) and histone H3 (N2). *C*) Three independent experiments from *B* were used to quantify the p65 protein amounts in the various fractions. The relative values were normalized to expression of the fraction markers, error bars show sem.

The cells expressing p65 and its DNA-binding and dimerization-defective variants were then analyzed for the kinetics of TNF-α–induced NF-κB signaling. The differentially reconstituted HeLa and MEF cells were treated for various periods with TNF-α and the kinetics of NF-κB activation was scored by the analysis of IκBα phosphorylation, decay and resynthesis. HeLa cells expressing p65 WT showed rapid IκBα phosphorylation and degradation upon short-term exposure to TNF-α, followed by resynthesis of IκBα after 60 min. In contrast, p65 E/I and p65 FL/DD expressing cells showed defective IκBα resynthesis in HeLa cells (**Fig. 3*A***) and MEFs (Supplemental Fig. S4*A*), comparable to the p65-deficient cells. HeLa cells expressing the p65 mutants also showed delayed or prolonged kinetics of TNF-α–induced p65 phosphorylation at Ser 468 and 536 (Fig. 3*A*), revealing that interference with DNA-binding or dimerization also affects access of kinases to the protein or even kinase-mediated signaling pathways. To compare the TNF-α–induced dynamic intracellular redistribution of p65 variants, cells were stimulated for various periods with TNF-α and fractionated into cytosolic (C), nucleoplasmic (N1) and chromatin (N2) extracts. In p65 WT cells, TNF-α caused the inducible and reversible translocation of p65 to the nucleoplasmic and chromatin fractions in HeLa cells (Fig. 3*B, C*) and MEFs (Supplemental Fig. S4*B*, *C*). In contrast, cells expressing p65 E/I showed a slightly prolonged residency time of p65 in the soluble nuclear (N1) fraction, consistent with the finding of an impaired IκBα synthesis. The p65 FL/DD mutant largely lost its ability for dynamic translocation to the nuclear fractions, suggesting that monomeric p65 cannot efficiently translocate to the nucleus.

### Expression of most TNF-α–induced NF-κB target genes depends on p65 DNA-binding

**Figure 4:**
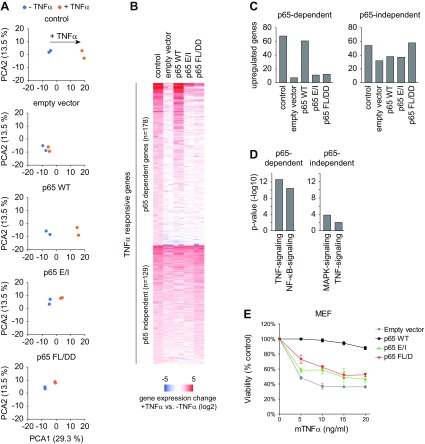
The role of p65 and its DNA-binding function for TNF-α–triggered gene expression. *A*) The indicated HeLa cell lines were treated for 1 h with TNF-α and the isolated RNA was analyzed by RNA-seq. A PCA analysis of the RNA-seq data in the indicated cell lines is displayed. *B*) The RNA-seq data were visualized in a heat map (in log_2_ scale) for TNF-α–regulated genes, which were grouped according to their dependency on p65 expression. *C*) The number of TNF-α–induced genes showing >2-fold regulation and their dependency from p65 function is displayed. *D*) The p65-dependent and -independent TNF-α–induced genes were analyzed for overrepresented pathways by using KEGG analysis, only the 2 top ranking pathways are displayed. *E*) Cell viability was assessed in p65^−/−^ MEFs reconstituted to express p65 WT, p65 E/I, or p65 FL/DD. Cells were exposed for 24 h to increasing concentrations of TNF-α and cell viability was measured. Experiments were performed in triplicates, and error bars represent sem.

TNF-α signaling dependent activation of gene transcription is facilitated by p65 activation, but also *via* p65-independent pathways, involving several MAPKs (46). To investigate the relative importance of p65 DNA-binding and dimerization for TNF-α–triggered gene expression, the differentially reconstituted HeLa and MEF cells were stimulated for 1 h with TNF-α, and gene expression was analyzed by RNA-seq experiments. A first data inspection by PCA further revealed that only some rudimentary functionality was still left in HeLa cells with DNA-binding or dimerization defective p65 compared to p65 WT cells (**Fig. 4*A***), showing the critical relevance of these features for p65-triggered gene expression.

We identified 307 genes that undergo dynamic TNF-α–dependent regulation based on a 2-fold threshold for regulation. From those genes ,more than half (178 genes) were dependent on NF-κB p65 (Fig. 4*B*), reinforcing the notion of NF-κB as a key master regulator of the inflammatory gene response (47). The vast majority of these p65-dependent genes were not regulated in the absence of functional DNA-binding (p65 E/I) or dimerization/DNA-binding (p65 FL/DD) (Fig. 4*C*). In this analysis, we also detected down-regulated genes, but they are not displayed because their number was too small to draw any statistically significant conclusions. The relevance of the DNA-binding and/or dimerization ability of p65 for TNF-α–induced expression of NF-κB target genes was also confirmed by reporter gene experiments (Supplemental Fig. S5*A*). To investigate the relevance of p65 for the induction of TNF-α–induced genes, a KEGG pathway analysis was performed to compare p65-dependent and -independent TNF-α–induced genes. As exemplified for the 2 top ranking biologic processes, p65-dependent genes are associated with the NF-κB signaling pathway, whereas the p65-independent genes show a more significant association with MAPK-signaling pathways (Fig. 4*D*). Thus, these data suggest that absence of p65 mainly impairs the p65-dependent gene activation but not the MAPK-dependent gene activation. Also, the RNA-seq analysis performed for TNF-α–stimulated p65-deficient and reconstituted MEFs revealed a very similar picture for the importance of p65 DNA-binding (Supplemental Fig. S5*B*) and the relative importance of NF-κB and MAPK-signaling pathways (Supplemental Fig. S5*C*). Taken together, these results show a predominant role of p65 itself and its DNA-binding and dimerization activity for the expression of a broad spectrum of TNF-α–regulated genes irrespective of the species and cell type. It was then interesting to test whether p65 DNA-binding and dimerization are also important for the physiologic antiapoptotic function of p65. Because TNF-α can kill MEFs deficient in NF-κB activating proteins (48), cells expressing WT or mutated forms of p65 were exposed to increasing concentrations of TNF-α and cell viability was measured. These experiments showed that inactivation of DNA-binding or dimerization sensitized MEF cells toward TNF-α–mediated cell death. The extent of these effects was largely comparable to p65-deficient cells (Fig. 4*E*).

### Chromatin association of p65 strongly depends on DNA-binding and dimerization

**Figure 5:**
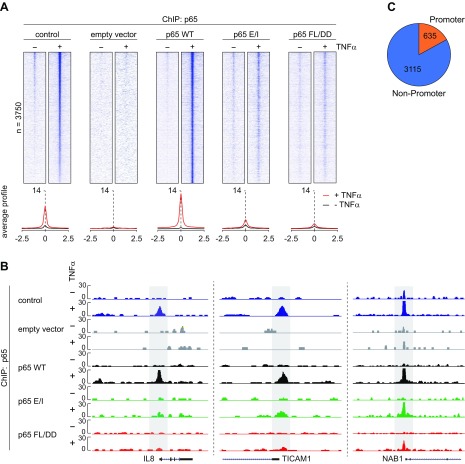
ChIP-seq analysis of TNF-α–induced chromatin binding of p65 and its DNA-binding and dimerization mutants. *A*) Upper: The indicated cell lines were stimulated for 1 h with TNF-α, and ChIP-seq experiments were performed by using an anti-p65 antibody. A ChIP-seq density heat map for p65 peaks is shown; color intensities represent normalized and globally scaled tag counts. The lower part shows the average p65 profiles for each investigated cell line before and after TNF-α treatment. *B*) Genome browser views of representative regions showing inducible DNA-binding of p65 WT and its mutants. The respective region highlighted in gray shows inducible p65 binding at promoters and transcribed genes. *C*) Pie chart showing the relative distribution of p65 binding to promoter and nonpromoter regions.

To investigate whether this lack of gene activation is due to impaired chromatin binding, we performed a ChIP-seq experiment in the various HeLa cell lines using a p65 antibody. HeLa control cells, p65 knockout cells, and the differentially reconstituted cells were stimulated for 1 h with TNF-α and genome association of p65 was determined by ChIP-seq experiments. Unstimulated HeLa control cells contained 861 constitutive p65 peaks, and their number increased to 3750 after TNF-α stimulation, as displayed in the peak density heat map shown in **Fig. 5*A***. These peaks were lost in p65-deficient cells and constitutive and TNF-α–induced chromatin recruitment was restored in cells reconstituted with the WT p65. In contrast, in cells expressing the p65 E/I or p65 FL/DD mutants, the p65 chromatin association was strongly diminished (Fig. 5*A*). These data prove the critical relevance of p65 DNA-binding and dimerization for chromatin binding to most, but not all, genomic loci. This is also illustrated at the example of specific genomic loci. Whereas the κB site in the *IL-8* promoter is only bound by the WT p65 protein, other NF-κB binding sites in the promoters controlling expression of *Ticam1* and *Nab1* still show recruitment of some p65 E/I or p65 FL/DD proteins (Fig. 5*B*). Most of the inducible p65 binding sites (3750 sites) are found outside from promoter regions that are only occupied at 635 sites (Fig. 5*C*), a distribution consistent with other studies (15, 36).

### Residual chromatin association of p65 mutants correlates with accessible chromatin structure and AP-1 binding

**Figure 6:**
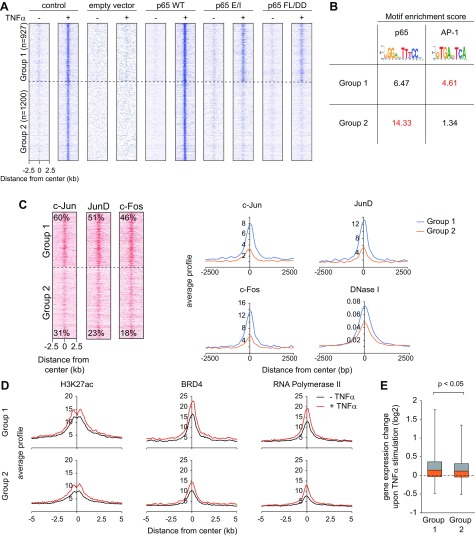
Genome-wide identification of p65 E/I and p65 FL/DD binding sites and their correlation with AP-1 binding and histone modifications. *A*) ChIP-seq density heat maps for regions with weak binding of both mutants (group 1) and no binding of neither of the mutants (group 2). *B*) Motif analysis of the 2 groups shows a distinct enrichment of motifs for p65 and AP-1. For each motif, the group where the motif is more strongly enriched is highlighted in red. *C*) The identified TNF-α–inducible p65-binding loci from group 1 and group 2 regions were compared to published binding profiles of the AP-1 TF subunits c-Jun, JunD, and Fos and DNase I accessibility. *D*) The identified TNF-α–inducible p65-binding regions from groups 1 and 2 were compared to published TNF-α–regulated profiles representing H3K27ac and binding of BRD4 and RNA polymerase II (36). Depicted are also the respective heat maps for c-Jun, JunD, and c-Fos. *E*) Gene expression change upon TNF-α stimulation of genes associated with the locations in group 1 or 2, respectively, is shown. The whisker plot shows the lower and upper quartile of the data with 5 and 95% whiskers. The *P* value was calculated by a 1-way ANOVA with Tukey’s *post hoc* test.

**Figure 7:**
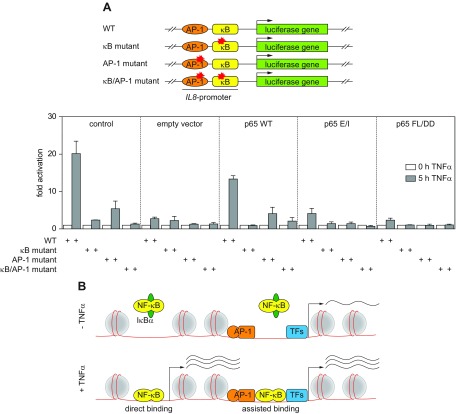
Functional analysis of AP-1–NF-κB interactions. *A*) The indicated HeLa cell lines were transfected with the different constructs encoding the Firefly reporter gene under the control of WT and mutant *IL-8* promoters together with a Renilla luciferase reporter that was used for normalization. After 2 d, the cells were stimulated for 5 h with TNF-α and luciferase activity was measured. Plot shows mean values of 4 individual experiments; error bars represents sem. *B*) A schematic model summarizing the proposed mechanisms for direct and assisted binding of p65 to the chromatin is shown.

To elaborate potential mechanisms that facilitate chromatin binding of the mutants, the TNF-α–inducible p65 binding sites were then classified in 2 opposing groups. Group 1 peaks (*n* = 927) show a residual TNF-α–triggered association of both p65 E/I or p65 FL/DD with chromatin, whereas group 2 peaks (*n* = 1200) lack any detectable binding activity of the p65 mutants (**Fig. 6*A***). For simplicity, binding sites that show an intermediate pattern between group 1 and 2 (*n* = 1623) were excluded from further analysis. Motif analysis showed an enrichment of the NF-κB DNA-binding motif at both groups. However, group 1 target sites have a lower normalized enrichment score for the NF-κB binding motif compared to group 2 target sites (Fig. 6*B*), implicating that those sites are less strictly dependent on the presence of the NF-κB binding motif. We hypothesized that other factors could contribute to p65 recruitment by supporting the chromatin binding of the mutant p65. Indeed, we observed that the binding motif for the heterodimeric TF AP-1 is more strongly enriched at group 1 targets (Fig. 6*B*). We confirmed the enrichment of the major AP-1 TFs c-Jun, JunD, and c-Fos at group 1 targets relative to group 2 targets using publicly available Encyclopedia of DNA Elements (ENCODE) data from HeLa cells (Fig. 6*C*) (35). This analysis shows that ∼50% of group 1 targets are bound by various members of the AP-1 protein family, whereas this association is strongly reduced at group 2 target sites. Group 1 targets also showed an increased DNase I hypersensitivity (Fig. 6*C*), suggesting that these loci possess a higher basal activity, which may enhance the accessibility for DNA-binding proteins such as p65. These data suggest the chromatin association of p65 mainly depends on its DNA-binding and dimerization function, but cooperativity with other factors may also contribute to p65 chromatin binding. This result is consistent with previous reports, demonstrating the cooperativity of p65 with other TFs, such as AP-1 (49, 50). Recent work demonstrated that TNF-α stimulation leads to a change of the chromatin landscape at p65 targets (36). Because the TNF-α–triggered transcriptional response is comparable between different cell types, we used the data set from the paper by Brown *et al.* (36) to address the question whether these 2 distinct groups of p65 targets differ in their ability to modulate the chromatin landscape in response to TNF-α. Interestingly, the enhancer mark H3K27ac, RNA polymerase II, and the acetyl-lysine binding BRD4 protein are more strongly enriched at group 1 target sites already under unstimulated condition (Fig. 6*D*), supporting our previous conclusion that group 1 sites possess higher basal activity. However, both groups show a similar increase of the investigated factors upon TNF-α stimulation, suggesting that p65 can activate transcription irrespective of its recruitment mechanism. Consistently, the associated genes showed a similar increase of their gene expression upon TNF-α stimulation, although genes related to group 1 targets were slightly more responsive (Fig. 6*E*). The functional interplay between AP-1 and NF-κB was further studied at the example of the TNF-α–responsive *IL-8* gene, which harbors binding sites for both TFs in its promoter region (50). The various HeLa cell lines were transfected with different luciferase reporter genes controlled by the *IL-8* promoter and harboring intact or mutated NF-κB and/or AP-1 binding sites. Cells were stimulated with TNF-α, and luciferase assays showed that TNF-α–induced gene expression is strongly reduced upon mutation of the AP-1 site and largely lost after mutation of the NF-κB or both binding sites (**Fig. 7*A***), consistent with previous findings in the IL-1 system (51). These experiments also revealed the full dependence on functional NF-κB for TNF-α–triggered *IL-8* expression, as evident by defective gene induction of the transiently transfected *IL-8* reporter gene in p65-deficient or p65-mutant HeLa cells (Fig. 7*A*). These experiments show the absolute requirement of NF-κB for *IL-8* expression and suggest that the reduced expression of *IL-8* upon mutation of the AP-1 binding site can be explained by its function as a cofactor for NF-κB.

In summary, these data indicate that NF-κB p65 DNA-binding is critical for most genomic binding sites, whereas residual chromatin association of the non-DNA–binding forms of p65 is likely enabled by protein-protein interactions with other TFs such as AP-1 in less compacted chromatin regions displaying elevated basal transcription. This finding supports the proposed mechanism that basal occupancy of TFs acts to prime chromatin and direct inducible TFs to selected regions in the genome (52).

## DISCUSSION

The DNA-binding activity of NF-κB p65 can be regulated by various PTMs, protein/protein interactions and by a number of small molecules. This work provides *in vivo* evidence that expression of most p65 target genes is fully dependent on the DNA-binding activity of p65 as the primary regulatory event. The functional consequences of p65-mediated transcription will then further be determined by the stoichiometry of TF heterodimers with TFs from other families (*e.g.*, AP-1) and also by the extent of p65 modifications. The DNA-binding capacity of p65 can also be compromised by disease-associated mutations of p65 residues with relevance for DNA-binding. For example, p65 E39 to Q mutations are found in lymphoid neoplasms (Cosmic database), suggesting that mutation of already 1 allele could lead to a significant reduction of NF-κB–driven transcription. Two different cell systems from 2 different species show that p65-dependent gene expression is almost completely compromised upon interference with p65 DNA-binding, suggesting that DNA-binding is of general importance for NF-κB function. However, p65 E/I mutants still showed some residual p65 chromatin attachment. These 2 different observations can be reconciled by the following considerations: First, not all p65 chromatin binding sites are controlling the expression of p65-dependent genes. Second, p65 mutant cells also show impaired expression of RelB, p100/p52, and probably also further transcriptional regulators, raising the possibility that defects in gene expression are also caused by inactivation of further gene regulatory proteins. Third, only high affinity binding of p65 might result in inducible gene expression.

Our work also emphasizes the relevance of p65 interaction with other TFs that occur at many gene regulatory regions. Direct binding of p65 has been described for the AP-1 family members c-Fos and c-Jun (49). Accordingly, a previous study showed that IL-1–inducible corecruitment of p65 and the AP-1 subunits c-Fos and JunD to the promoters of the NF-κB target genes *IL-8* and *CXCL2* was dependent on the presence of the p65 protein (15). The auxiliary function of AP-1 binding for strictly NF-κB–dependent expression of the *IL-8* gene was also found in this study after mutation of the AP-1 binding site. Conversely, in murine fibroblasts, c-Jun was required for constitutive and inducible chromatin recruitment of p65 to the *Ccl2* locus (53). Cooperation of NF-κB has also been observed with further TFs including SP1 (54), STAT3 (55), Retinoid X receptor, α (56), and the glucocorticoid receptor (57). The cooperation between p65 and further TFs has also been revealed by ChIP-seq experiments that showed overlapping genomic association between p65 and other TFs such as E2F1 (58) and the forkhead box protein FOXM1 (59). The local clustering of TFs does not only increase the local density of transactivation domains to foster gene expression but also allows mutual assistance for DNA-binding in the chromatin context. This is exemplified by p65-assisted IRF5 chromatin-binding in LPS-stimulated cells (60). The interactions between TFs also contribute to the selection of the appropriate DNA-binding site among the myriad of potential estimated >300,000 consensus NF-κB genomic binding sites (61). The group 1 sites still allowing residual TNF-α–triggered association of p65 E/I are characterized by increased occupation with AP-1 subunits and accessible chromatin. This situation is reminiscent to the glucocorticoid receptor, which requires the AP-1–dependent maintenance of baseline chromatin accessibility to allow hormone-dependent glucocorticoid receptor recruitment (52). This is consistent with the proposed model that basal occupancy of TFs acts to prime chromatin for the subsequent association of inducible TFs (62). Such a mechanism would also ensure the expression of a subset of genes without prior chromatin remodeling, consistent with an earlier observation describing the association of a fraction of p65, with regions showing already activation marks such as H3K4me1 and H3K27ac in unstimulated cells (63). Such a mechanism is schematically displayed in Fig. 7*B* and could ensure the rapid mounting of an immune response without any time delays caused by chromatin remodeling processes. This rapid mounting of the immune response is needed to combat infections with exponentially growing bacteria or viruses.

## Supplementary Material

This article includes supplemental data. Please visit *http://www.fasebj.org* to obtain this information.

## Abbreviations

AP-1: activator protein 1
ChIP: chromatin immunoprecipitation
ChIP-seq: ChIP with massively parallel DNA sequencing
HA: hemagglutinin
MEF: mouse embryonic fibroblast
PCA: principal component analysis
PTM: posttranslational modification
RNA-seq: RNA-sequencing
TF: transcription factor
WT: wild type
