# Supplementary material for: NF-κB p65 dimerization and DNA-binding is important for inflammatory gene expression
Source: FASEB J. 2018 Dec 7;33(3):4188–202. doi: 10.1096/fj.201801638R (PMC6404571; doi:10.1096/fj.201801638R)
Supplement: Supplementary file 1 [file fj.201801638R.sf1.pdf]

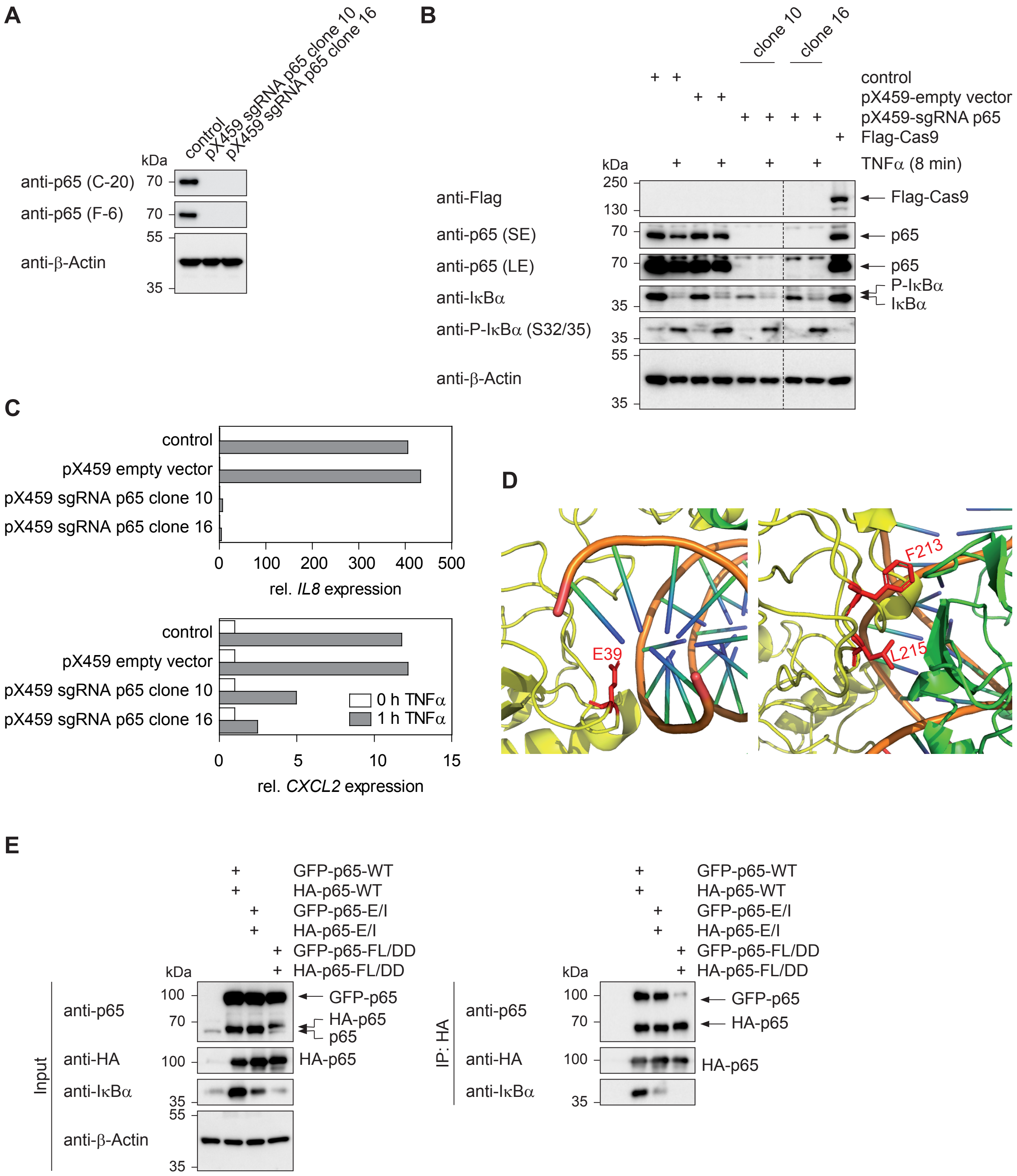

Riedlinger et al. Suppl. Fig. 1

Suppl. Fig. 1. Generation and characterization of p65 mutant cell lines. (A) HeLa cells were transfected with the vector pX459-hp65 encoding a p65-specific sgRNA or the empty vector control. Transfected cells were selected by puromycin treatment and surviving clones were grown to colonies. Expression of p65 was detected with antibodies recognizing the C-terminus (C-20) and the N-terminus (F-6) in control cells and knockout clones # 10 and 16. **(B)** The indicated control and p65-deficient cell clones were stimulated for 8 min with TNFα, followed by immunoblotting for the analysis of expression levels of p65, Flag-Cas9 and β-Actin with specific antibodies. In addition, also the levels and phosphorylation of IκBα were determined by immunoblotting as shown. The p65 protein was detected in a short exposure (SE) and long exposure (LE). **(C)** The indicated control and knockout cell lines were exposed for 1 h to TNFα and expression of the indicated NF-κB target genes was analyzed by qPCR with specific primers, a representative experiment is shown. **(D)** The dimer between p65 (yellow) and p50 (green) in complex with DNA is shown (PDB code 1vkx) (65). The Pymol program was used to highlight the mutated Glu 39 in red (left). The second picture displays a top view on the positions of Phe 213 and Leu 215 within the dimerization domain (right). **(E)** HEK293T cells were transfected to express GFP- and HA-tagged p65 in the wildtype or mutated forms as shown. Cell extracts were generated and used for Western blotting (left) or for co-immunoprecipitation experiments using anti-HA-agarose (right) as shown. Molecular weight markers are given, the positions of the detected proteins are indicated by arrows.
