# Supplementary material for: NF-κB p65 dimerization and DNA-binding is important for inflammatory gene expression
Source: FASEB J. 2018 Dec 7;33(3):4188–202. doi: 10.1096/fj.201801638R (PMC6404571; doi:10.1096/fj.201801638R)
Supplement: Supplementary file 3 [file fj.201801638R.sf3.pdf]

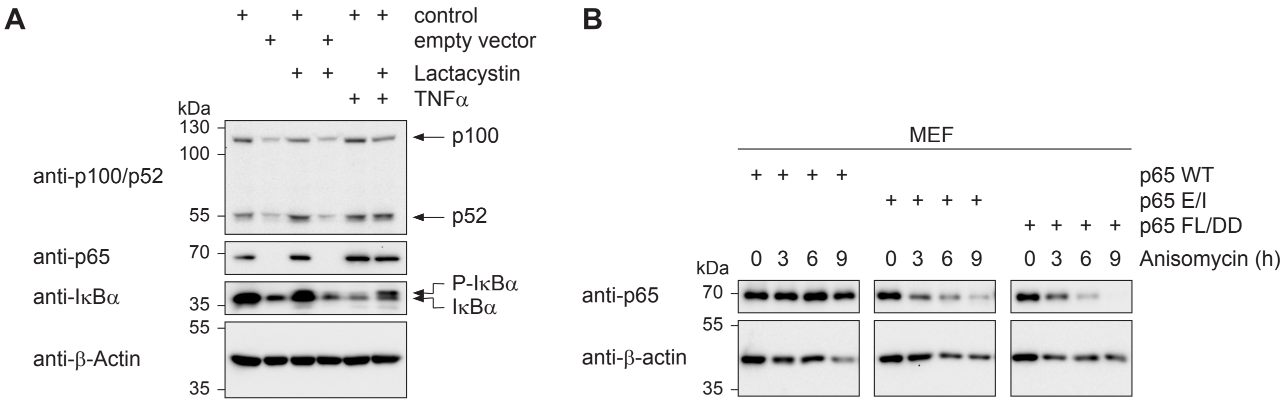

Riedlinger et al. Suppl. Fig. 3

Suppl. Fig. 3. Functional analysis of p65 FL/DD stability and diminished p100 expression in p65-deficient cells. (A) Control HeLa cells and HeLa p65 knockout clone #10 cells retransfected with an empty vector were treated with Lactacystin (10 μM) for 4 h and with TNFα for 15 min as shown. Cell extracts were prepared and analyzed for abundance of p100/p52 and IκBα as shown. The position of phosphorylated IκBα is indicated, the proteasome inhibiting effect of Lactacystin is seen by stabilization of IκBα in TNFα-treated cells. **(B)** The different MEF cell lines were treated for the indicated periods with the protein synthesis inhibitor anisomycin (2,5 μg/ml). The amounts of p65 and β-Actin were revealed by immunoblotting.
