# Supplementary material for: NF-κB p65 dimerization and DNA-binding is important for inflammatory gene expression
Source: FASEB J. 2018 Dec 7;33(3):4188–202. doi: 10.1096/fj.201801638R (PMC6404571; doi:10.1096/fj.201801638R)
Supplement: Supplementary file 4 [file fj.201801638R.sf4.pdf]

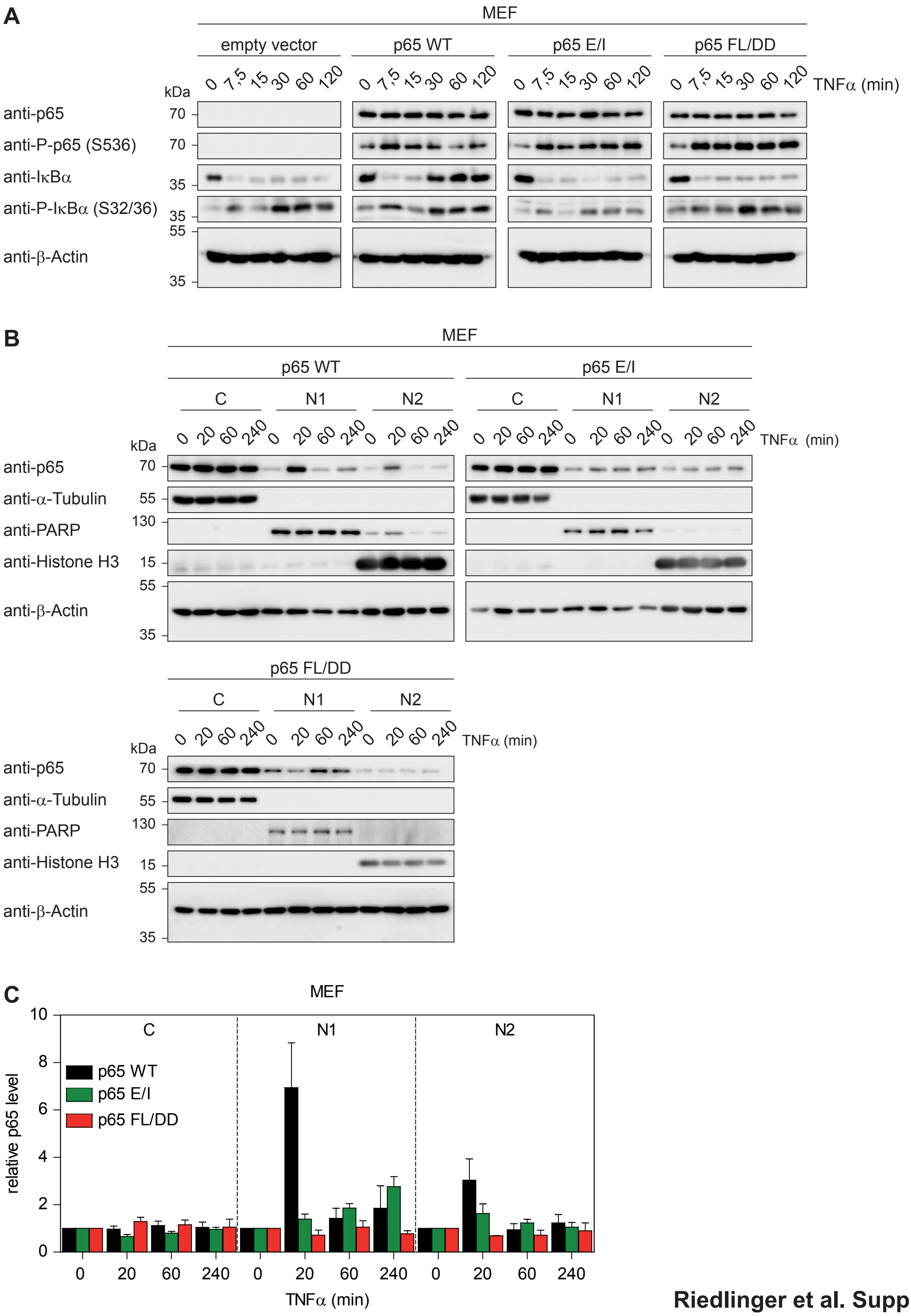

Riedlinger et al. Suppl. Fig. 4

Suppl. Fig. 4. Analysis of differentially reconstituted MEF cells for changes in dynamic NF-κB signaling. (A) The reconstituted MEFs cells were stimulated for different periods with TNFα and stability and phosphorylation of p65 and IκBα was analyzed by immunoblotting, a representative experiment is displayed. **(B)** The differentially reconstituted MEFs were treated for the indicated periods with TNFα and cells were fractionated into the cytosolic (C), soluble nuclear (N1) and insoluble nuclear (N2) fractions. These fractions were analyzed for the kinetics of p65 nuclear import and export by immunoblotting. The purity of the fractions was controlled by blotting for Tubulin (C), PARP (N1) and histone H3 (N2). **(C)** Three independent experiments from (B) were used to quantify the p65 protein amounts in the various fractions. The relative values were normalized to expression of the fraction markers, error bars show standard error of the mean.
