# Supplementary material for: NF-κB p65 dimerization and DNA-binding is important for inflammatory gene expression
Source: FASEB J. 2018 Dec 7;33(3):4188–202. doi: 10.1096/fj.201801638R (PMC6404571; doi:10.1096/fj.201801638R)
Supplement: Supplementary file 5 [file fj.201801638R.sf5.pdf]

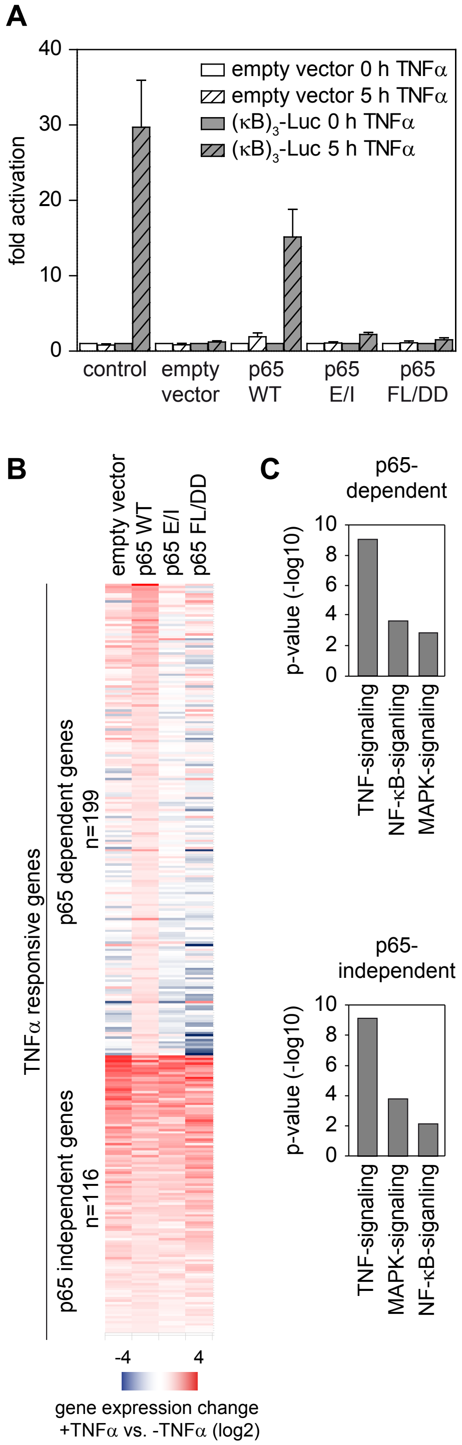

Riedlinger et al. Suppl. Fig. 5

Suppl. Fig. 5. The role of p65 and its DNA-binding function for TNFα-triggered gene expression in HeLa and MEF cells. (A) The indicated reconstituted HeLa cell lines were transfected with a NF-κB-dependent Firefly luciferase reporter gene and a construct allowing the expression of Renilla luciferase under the control of a constitutive promoter. Two days later the cells were stimulated as shown and luciferase activity was determined. Plot shows mean values of four individual experiments, error bars show standard error of the mean. **(B)** The indicated MEF cell lines were treated for 1 h with TNFα and the isolated RNA was analyzed by RNA-seq. The RNA-seq data were visualized in a heat map (in log2 scale) for TNFα-regulated genes, which were grouped according to their dependency on p65 expression. **(C)** The p65-dependent and -independent TNFα-induced genes were analyzed for overrepresented pathways using KEGG analysis, only the three top ranking pathways are displayed.
