# Supplementary material for: NF-κB p65 dimerization and DNA-binding is important for inflammatory gene expression
Source: FASEB J. 2018 Dec 7;33(3):4188–202. doi: 10.1096/fj.201801638R (PMC6404571; doi:10.1096/fj.201801638R)
Supplement: Supplementary file 6 [file fj.201801638R.st1.docx]

**Riedlinger et al. supplementary table 1**

**Antibodies**

| **Primary antibody (clone)** | **Species** | **Supplier** |
| --- | --- | --- |
| p65 (C-20) | rabbit pAb | Santa Cruz #sc-372 |
| p65 (F-6) | mouse mAb | Santa Cruz #sc-8008 |
| P-p65 (S536) (9BH1) | rabbit mAb | Cell Signalling #3033 |
| P-p65 (S468) | rabbit pAb | Cell Signalling #3039 |
| RelB (D-4) | mouse mAb | Santa Cruz #sc-48366 |
| c-Rel (B-6) | mouse mAb | Santa Cruz #sc-6955 |
| p50/105 (NLS) | rabbit pAb | Santa Cruz #sc-114 |
| p52/100 (C-5) | mouse mAb | Santa Cruz #sc-7386 |
| IκBα (C21) | rabbit pAb | Santa Cruz #sc-371 |
| P- IκBα (S32/26) (5A5) | mouse mAb | Cell Signalling #9246 |
| P- IκBα (S32) (14D14) | rabbit mAb | Cell Signalling #2859 |
| IκBβ (C-20) | rabbit pAb | Santa Cruz #sc-945 |
| IκBε (M121) | rabbit pAb | Santa Cruz #sc-7156 |
| β-Actin | rabbit pAb | Abcam #ab8227 |
| α-Tubulin | mouse mAb | DSHB #12G10 |
| PARP (C-2-10) | mouse mAb | Clontech #630210 |
| Histone H3 | rabbit pAb | Abcam #ab1791 |
| Flag (M2) | mouse mAb | Sigma Aldrich #F3165 |
| GFP | mouse mAb | Roche #11814460001 |
| HA (3F10) | rat mAb | Roche #11867423001 |
| Normal Rabbit IgG | rabbit | Cell Signalling #2729S |

| **Secondary antibody (clone)** | **Conjugated to** | **Supplier** |
| --- | --- | --- |
| goat-anti-mouse IgG | HRP | Dianova #112-035-143 |
| goat-anti-rabbit IgG | HRP | Dianova #111-035-144 |
| goat-anti-rat IgG | HRP | Dianova #112-035-143 |

**Plasmids**

| **Plasmid** | **Origin** | **Reference** |
| --- | --- | --- |
| pX459 | F. Zhang | PMID: 24157548 |
| pX459-sgRNA hu p65 | M.L. Schmitz | this study |
| pEF-Puro-HA (empty vector) | M.L. Schmitz | PMID: 16407239 |
| pEF-Puro-hu p65 WT-HA | M.L. Schmitz | PMID: 16407239 |
| pEF-Puro-hu p65 E/I-HA | M.L. Schmitz | this study |
| pEF-Puro-hu p65 FL/DD-HA | M.L. Schmitz | this study |
| pFG9-EF1α-Puro (empty vector) | E. Burstein | PMID: 21706061 |
| pFG9-EF1α-Puro-mp65 WT-HA | E. Burstein | PMID: 21706061 |
| pFG9-EF1α-Puro-mp65 E/I-HA | M.L. Schmitz | this study |
| pFG9-EF1α-Puro-mp65 FL/DD-HA | M.L. Schmitz | this study |
| pcDNA3.1-p65 WT-GFP | M.L. Schmitz | PMID: 16407239 |
| pcDNA3.1-p65 E/I-GFP | M.L. Schmitz | this study |
| pcDNA3.1-p65 FL/DD-GFP | M.L. Schmitz | this study |
| pCR3 FLAG-hu RelB | S. Hailfinger | PMID: 21873235 |
| (κB)_3_-Luc | M.L. Schmitz | PMID: 9660769 |
| pUHC13-3-*Il8* prom-luc WT | M. Kracht | PMID: 10490613 |
| pUHC13-3-*Il8* prom-luc κB-mut | M. Kracht | PMID: 11071890 |
| pUHC13-3-*Il8* prom-luc AP-1 mut | M. Kracht | PMID: 10490613 |
| pUHC13-3-*Il8* prom-luc κB+AP-1 mut | M. Kracht | PMID: 11050078 |
| pCI-Renilla luc | M.L. Schmitz | PMID: 28615693 |

**DNA-Oligonucleotides**

| **Oligo name** | **Sequence (5´ to 3´)** |
| --- | --- |
| px459-hu p65-for | CACCGCTTCCGCTACAAGTGCGA |
| px459-hu p65-rev | AAACTCGCACTTGTAGCGGAAGC |
| hu CXCL2-for | AGCTTGTCTCAACCCCGCATC |
| hu CXCL2-rev | GGGCAGGGCCTCCTTCAGG |
| hu IL8-for | AGTGGACCACACTGCGCCAA |
| hu IL8-rev | TCTCCACAACCCTCTGCACC |
| NF-κB EMSA for | AGTTGAGGGGACTTTCCCAGGC |
| NF-κB EMSA rev | GCCTGGGAAAGTCCCCTCAACT |

**Reagents**

| **Name** | **working concentration** | **Supplier** |
| --- | --- | --- |
| human TNFα | 20 ng/ml | ImmunoTools #11343015 |
| murine TNFα | 20 ng/ml | ImmunoTools #12343014 |
| Anisomycin | 2,5 µg/ml | Sigma-Aldrich #A9789 |
| Lactacystin | 10 µM | Enzo Lifesciences #BML-PI104-0200 |
| Monoclonal Anti-HA Agarose |  | Sigma Aldrich # A 2095 |
| GFP-trap |  | Chromotek # gta-20 |
|  |  |  |
