# Supplementary material for: NF-κB p65 dimerization and DNA-binding is important for inflammatory gene expression
Source: FASEB J. 2018 Dec 7;33(3):4188–202. doi: 10.1096/fj.201801638R (PMC6404571; doi:10.1096/fj.201801638R)
Supplement: Supplementary file 2 [file fj.201801638R.sf2.pdf]

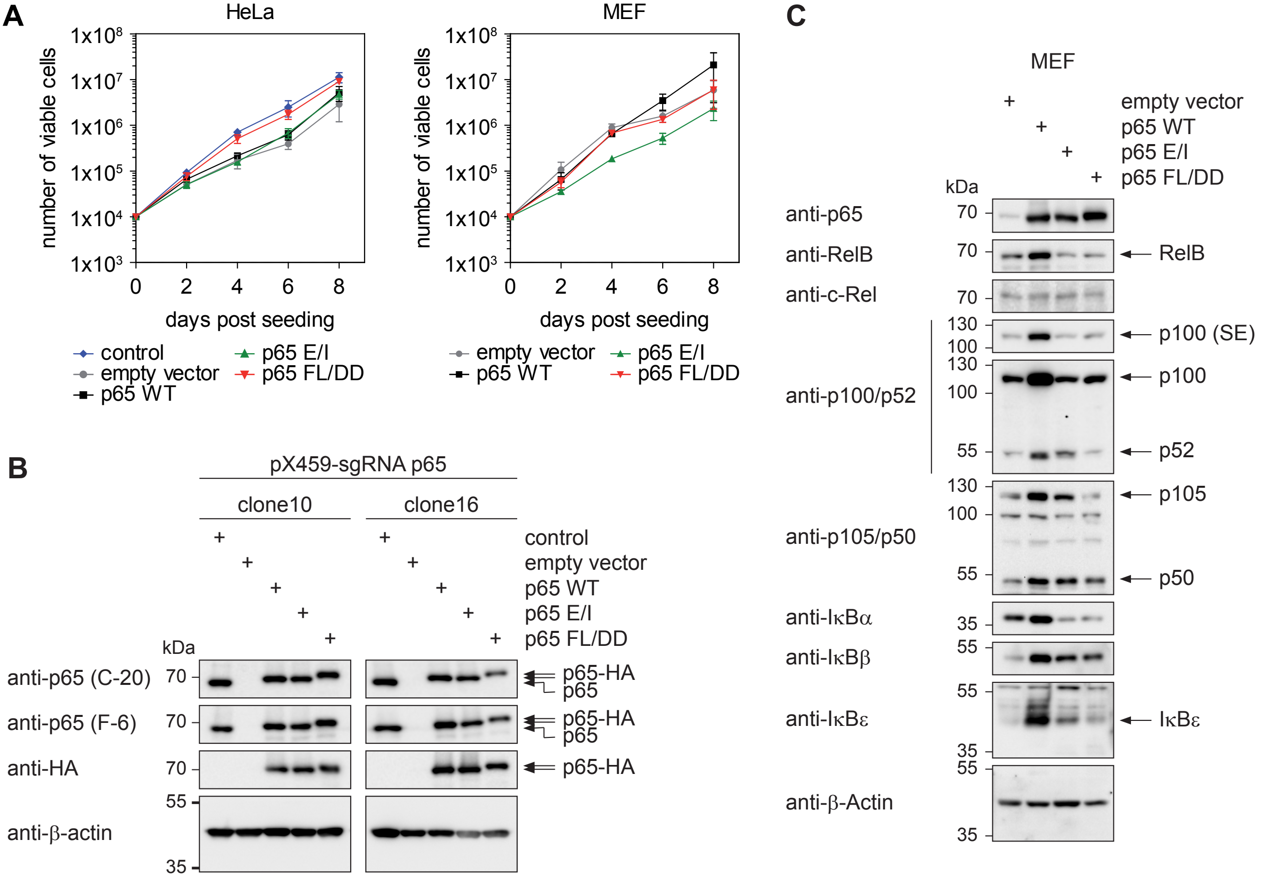

Riedlinger et al. Suppl. Fig. 2

Suppl. Fig. 2. Characterization of p65 mutant cell lines. (A) Equal numbers of cells from the indicated HeLa (left) and MEF (right) cell lines were seeded and their proliferation was monitored for 8 days, mean values from 3 experiments are shown, error bars show standard error of the mean. **(B)** The indicated cell HeLa p65 knockout clones # 10 and 16 were used for the generation of stable cell lines expressing the indicated p65 proteins containing a C-terminal HA tag. **(C)** NF-κB p65$^{-/-}$ MEFs were used for the generation of stable cell lines expressing physiological amounts of p65 and its mutants. Expression of p65 and other NF-κB regulatory proteins were assessed by immunoblotting as indicated.
